# Animal health is often ignored, but indispensable to the human right to health

**DOI:** 10.1186/s12939-021-01613-0

**Published:** 2022-01-10

**Authors:** Sonja Hartnack

**Affiliations:** Section of Epidemiology, Vetsuisse Faculty, University of Zurich, Winterthurerstr. 270, 8057 Zurich, Switzerland

**Keywords:** One health, Rabies, Dogs, Mass vaccination, Responsible dog ownership

## Abstract

Although preventable by vaccines, approximately 60′000 humans die due to canine transmitted rabies annually, mostly in Africa and Asia. The aim of this paper is to advocate for including animal health aspects into considerations of human health and human rights, and for equitable access to rabies vaccination for both animals and humans. An infringement of human - in particular of children’s - right to health will be illustrated with the case of rabies and poor dog management in Uganda.

## Background

### Rabies vaccination and post-exposure prophylaxis

Although preventable by vaccines, approximately 60′000 human rabies deaths occur annually, mostly in Africa and Asia [1].

Since “canine rabies mostly affects the poorest sectors of society in the world’s poorest countries”, in combination with rabies being a fatal disease, lack of access to treatment and deaths at home, outside the health systems mean that cases are not recorded in surveillance systems based on health records [2], leading to underreporting of human rabies cases. Thus, global inequities in access to preventive rabies vaccination have led to the persistence of the rabies problem in lower income settings. Out of pocket payment required for vaccination and treatment leads to local inequities, which affect those at highest risk.

The majority of human deaths due to rabies occur in children aged 5–14 years old. Domestic dogs contribute up to 99% of all rabies transmissions to humans [3]. Treating a rabies exposure, where the average cost of rabies post-exposure prophylaxis (PEP) is US$ 40 in an African country like Uganda, can be a catastrophic financial burden for a family whose average daily income is around US$ 1–2 per person (https://dataafrica.io/profile/uganda#poverty, accessed 11 December 2021). Furthermore, PEP is not always available or accessible for those in need. It has been shown that dog vaccination is cost-effective in preventing human rabies, compared to control strategies focussing solely on PEP. For example in Latin America, implementing mass dog vaccinations led to a 90% reduction of human deaths due to rabies since 1983 [4]. This commentary describes the situation of rabies and prevailing inequities and factor which contribute to human deaths due to canine transmitted rabies in Uganda.

### Rabies in Uganda

Nearly 90% of the population in Uganda live in canine rabies endemic areas [5]. Canine vaccines are much cheaper (US$ 1) than PEP and are (in theory) available in most Ugandan districts. However, distribution and administration of canine rabies vaccines are hampered by a number of logistical factors, e.g. lack of transport, maintenance of the cold chain, and facilitation of village health teams, and lack of data on dog population size (no mandatory registration of dogs) for efficient planning for canine mass vaccination.

Furthermore in Uganda, the laboratory capacity to diagnose canine rabies is only possible in two laboratories: the National Animal Disease Diagnostic and Epidemiology Center, NADDEC in Entebbe, and the Central Diagnostic Laboratory at the College of Veterinary Medicine, Animal Resources and Biosecurity, Makerere University, Kampala. but Transport of samples from remote regions is difficult, expensive, and results are often only available after a few weeks, which is too late to impact clinical care. A scarcity in PEP in rural areas has been described [6]. When there is no opportunity to confirm or exclude a rabies diagnosis in a dog who has bitten a child, unnecessary PEP might use resources which could be better allocated somewhere else in an underfinanced health care system and impose catastrophic health expenditure on families. All of these logistical inequities have contributed to the ongoing rabies burden in Uganda which has practical and ethical consequences for dogs, communities and veterinarians.

Although not advisable from an epidemiological perspective, mass culling of free roaming dogs with strychnine - or other inhumane techniques - is still undertaken in Uganda. Given the absence of laboratory capacity for confirmatory diagnosis of rabies in most parts of the country, it is difficult to tell whether a dog died due to rabies or strychnine. This culling procedure is ethically questionable in that healthy dogs may be killed. Non-targeted animals (dogs and other species, including wild animals), children and some humans who scavenge around waste disposal sites where such poisons are usually laid may also be at risk of poisoning. This culling procedure causes moral distress in veterinarians, and additionally precludes obtaining reliable data on the number of rabid dogs. From an epidemiological perspective is mass culling of dogs is not advisable as in a short time the killed dogs will be replaced with other dogs, possibly introducing rabies again.

### One health and surveillance

A lack of surveillance and of accurate data, both in humans (cases of rabies and of dog bites) as well as in animals, has rendered rabies a low public health and veterinary priority in most rabies endemic countries. A lack of inter-sectoral collaboration, as recommended in the One Health framework, is a key factor hampering effective rabies control. In the current existing electronic surveillance (IDSR, Integrated Disease Surveillance and Response) data on human dog bite victims are recorded, but there is no information recorded about the dog and its vaccination status, nor does the district veterinary office has a direct link to IDSR. Anecdotally, human dog bite victims present themselves at the district veterinary office instead of the health center. One Health recognizes that the health of humans, animals and ecosystems are interconnected. It involves applying a coordinated, collaborative, multidisciplinary and cross-sectoral approach to address potential or existing risks that originate at the animal-human-ecosystems interface (http://www.onehealthglobal.net/, accessed 11 December 2021).

Thus, almost all human deaths due to rabies can be understood as a failure of the health.

system [7], both human public health and veterinary public health, as a consequence of the prevailing spectrum of inequities. After the introduction of policy reforms in 1993 in Uganda, local government veterinary personnel was reduced, contagious and other major animal diseases increased by 46% and field animal vaccine availability decreased by 64.3% [8]. The poorly performing and underpowered veterinary and public health services, as well as the neglect by administration and government is in stark contravention [9] of the Rights of the Child.

(https://www.unicef.org/child-rights-convention/convention-text, accessed 11 December 2021) proclaiming that “*States Parties shall ensure to the maximum extent possible the survival and development of the child*”.

In the case of human rabies and large-scale control measures have been implemented since 1920 (reviewed in [10]). A number of international organisations and initiatives, i.e. World Health Organization (WHO), Food and Agriculture Organization of the United Nations (FAO), World Organisation for Animal Health (OIE) and the Global Alliance for Rabies control (GARC), have agreed on a country-driven strategic target of achieving “zero human deaths due to canine rabies in 2030”.

(https://www.who.int/rabies/Executive_summary_draft_V3_wlogo.pdf?ua=1, accessed 11 December 2021).

### Canine and human health

Responsible dog ownership, including access to food and basic veterinary care (including vaccinating against rabies, parvovirosis, canine distemper and deworming) to reduce the risk of Rabies is beneficial in terms of the human right to health. Next to a decrease in dog bites, healthy vaccinated dogs will live longer, the effect of mass vaccination will also last longer and there will be less transmission of zoonotic diseases. It has been estimated that if 70% of the dog population is vaccinated, rabies can be prevented or even eliminated [11]. At present, the life of rural dogs in Western Uganda has been described as “Hobbesian”, namely poor, nasty, brutish, and short [12]. A stable dog population is therefore beneficial for human health and dogs. Additionally, dog population control by neutering or spaying is a useful preventive measure to avoid an increasing number of roaming dogs. Thus in the case of rabies, including the welfare of dogs as part of appropriate rabies control measures is deemed to be indispensable for the achievement of the human health goal of “zero human deaths due to canine transmitted rabies” and is in line with the human - particularly children’s – right to health.

## Conclusion

To pave the way for elimination of rabies by 2030 a multifaceted approach is needed, involving a) better surveillance of the human bite victims and the suspected rabid dog including its vaccination status and data exchange between the human and animal health sector, b) higher canine rabies vaccination coverage, c) appropriate treatment of bite victims (wound cleaning and PEP administration) and d) increased laboratory capacities for robust and timely test results in decentralised labs e) sensitisation and training at community level, incl. Teacher, children and dog owner on rabies and dog bite prevention and f) fostering responsible dog ownership for better human health. An example for harnessing community action is given by Isiko et al. (2017) [13]. There is a need for recognition that improving equity in access to rabies vaccination will have many benefits for the public and the individual affected by rabies disease, and will contribute to reducing moral distress among the veterinarians who are those currently having to deal with the problems created by the vaccine inequities. It is time to consider health as a universal good, relevant for humans, animals, and the environment. Promoting the health of animals and the environment is crucial to safeguard human health [14].

## Data Availability

Not applicable.
